# Genes with a Combination of Over-Dominant and Epistatic Effects Underlie Heterosis in Growth of *Saccharomyces cerevisiae* at High Temperature

**DOI:** 10.3389/fgene.2016.00072

**Published:** 2016-05-04

**Authors:** Rachel Shapira, Lior David

**Affiliations:** Department of Animal Sciences, R. H. Smith Faculty of Agriculture, Food and Environment, The Hebrew University of JerusalemRehovot, Israel

**Keywords:** over-dominance, hybrid vigor, QTL, epistasis, quantitative traits, yeast, genetic interaction

## Abstract

Heterosis describes a phenotypic phenomenon of hybrid superiority over its homozygous parents. It is a genetically intriguing phenomenon with great importance for food production. Also called hybrid-vigor, heterosis is created by non-additive effects of genes in a heterozygous hybrid made by crossing two distinct homozygous parents. Few models have been proposed to explain how the combination of parental genes creates an exceptional hybrid performance. Over-dominant mode of inheritance is an attractive model since a single gene can potentially create the heterotic effect, but only a few such loci have been identified. To a collection of 120 hybrids, made by crossing 16 divergent *Saccharomyces cerevisiae* yeast strains, we applied a method for mapping heterozygous loci that non-additively contribute to heterotic growth at 37°. Among 803 candidate loci that were mapped, five were tested for their heterotic effect by analyzing backcrosses and F2 populations in a specific hybrid background. Consistently with the many mapped loci, specific analyses confirmed the minor heterotic effect of the tested candidate loci. Allele-replacement analyses of one gene, *AEP3,* further supported its heterotic effect. In addition to over-dominant effects, the contribution of epistasis to heterosis was evident from F2 population and allele-replacement analyses. Pairs of over-dominant genes contributed synergistically to heterosis. We show that minor over-dominant effects of multiple genes can combine to condition heterosis, similarly to loci affecting other quantitative traits. Furthermore, by finding of epistatic interactions between loci that each of them individually has an over-dominant effect on heterosis, we demonstrate how hybrid advantage could benefit from a synergistic combination of two interaction types (over-dominant and synergistic epistatic). Thus, by portraying the underlying genetic complexity, these findings advance our understanding of heterosis.

## Introduction

The phenomenon describing superiority of heterozygous progeny over their homozygous parents is called heterosis or hybrid vigor (East, 1908; Shull, 1908, 1911). Incorporating crossbreeding strategies to improve production in crop plants and livestock often resulted in heterosis that advanced dramatically global food production. Despite its practical significance and the long-standing curiosity in the genetics of heterosis, the complexity of this phenomenon renders its genetic and molecular bases challenging to uncover. Various genetic models provide explanations for how heterozygosity in hybrids might contribute to their performance advantage over their homozygous parents.

From a genetic point of view, the three main models that explain heterozygote advantage rely on dominance or deviation from additive effects in one or more loci (Lippman and Zamir, 2007). The dominant complementation model proposes that dominant alleles from both parents reciprocally complement negative effects of recessive alleles, creating a hybrid with less recessive alleles than either parent (Bruce, 1910). Another model explains how interactions between alleles of different loci, epistasis, which are created due to new combinations in the hybrid, contribute to its improved performance (Crow, 1948; Comstock and Robinson, 1949; Comstock et al., 1949). A third model explains heterosis by synergistic effects of over-dominant interactions between alleles created by a heterozygous state within one or more loci (East, 1908; Shull, 1908; Crow, 1948). Additionally, a few more models, which do not necessitate dominance, explain heterosis by an overall improvement in hybrid's metabolism, energy production, and energy utilization that could result from gene dosage effects, polyploidy, changes in gene expression levels and changes in function of protein complexes (Hedgecock et al., 2007; Baranwal et al., 2012; Schnable and Springer, 2013).

The level of superiority varies considerably among hybrids and conditions, making heterosis a quantitative phenomenon. This phenomenon can often be found in reproductive fitness traits that are quantitative by themselves, like some of the production traits in plants and animals. Therefore, methods used to map loci contributing to heterosis were similar to those used in mapping quantitative trait loci (QTL; Deutschbauer and Davis, 2005; Altshuler et al., 2008; Mackay et al., 2009). Unlike regular QTL mapping studies, mapping QTL contributing to heterosis is actually mapping the mode of inheritance of contributing loci rather than the contribution alone. It is conceivable that if heterosis shares similarities with other quantitative traits, the genetic basis underlying the superior performance of the hybrid will be composed of many loci, each with a minor or mild dominant effect due to interactions between alleles within and between loci. As for many other quantitative traits, also for heterosis, loci with minor effects are harder to identify than loci with major effect.

While many studies mapped loci contributing to heterosis, identifying over-dominant loci turned out to be a more difficult task. Consequently, there are only a few known cases, mostly in plants, of single genes that confer heterotic effect by an over-dominant interaction between their alleles (Hua et al., 2003; Krieger et al., 2010; Saeed et al., 2011; Li et al., 2013). Over-dominant loci are interesting because of the nature of interaction between their alleles that confers a synergistic effect. Furthermore, for these known over-dominant loci, a major phenotypic contribution was measured, making such loci attractive and feasible to utilize for improving performance of hybrids in traits of interest. On the other hand, some loci that were initially mapped as over-dominant were later studied further and turned out to be pseudo over-dominance loci. In pseudo over-dominance cases, two or more genetically linked genes, each having a dominance complementation effect but in repulsion phase, give the impression of one over-dominant QTL. The existence of pseudo over-dominance loci is used to argue against the prevalence estimates of true over-dominance loci that were obtained in some heterosis QTL studies (Charlesworth and Willis, 2009; Schnable and Springer, 2013).

We have constructed a collection of 120 heterozygous hybrids by crossing 16 homozygous parental strains and measured their growth rate in five environmental conditions (Shapira et al., 2014). In this collection of hybrids, we identified a significant amount of best-parent heterosis. Thirty five percent of hybrid-condition combinations were heterotic, a large proportion that makes this collection suitable for studying the genetic basis of heterosis. Analyses of phenotypic results provided evidence that all three genetic models, namely, dominant complementation, over-dominance, and epistasis, could be used to explain cases of heterosis found in this collection. Since the mechanisms underlying the synergistic effect of over-dominant loci are not well-understood, in this study we used the yeast model to identify such loci and characterize their contribution.

## Materials and methods

### Growth media and strains

YEPD rich medium [1% yeast extract (BD Bacto), 2% peptone (BD Bacto), 2% dextrose (J. T. Baker)] was used unless other specific medium is mentioned. For selection, YEPD was supplemented with 300 mg/L Hygromycin B (InvivoGen), 400 mg/L G418—Geneticin (Santa Cruz) or both. For selecting Δ*URA3* strains, Synthetic Complete (SC) medium was prepared with 5-FOA [2% dextrose, 0.17% yeast nitrogen base (Difco), 0.14% amino acid dropout, 0.5% ammonium sulfate (Sigma), 0.08% 5-Fluoroorotic acid (US Biological) and the required amino acids]. SC medium lacking uracil was used for selecting cells with intact *URA3*. Solid media were prepared by addition of 2% agar (Difco) to any of the liquid media listed above. The strains used in this study are diallel strains (Shapira et al., 2014) and manipulated strains as described in the text that were made in two genetic backgrounds: S288c (Winzeler et al., 1998) and SK1(Kane and Roth, 1974).

### Sporulation of diploids and tetrad dissection

Diploid cells were grown on YEPD plates in patches overnight and scraped off from the plates into sporulation solution [0.75% potassium acetate (Sigma)] for 2–3 days. To prepare for dissection, an aliquot of 15 μL from the sporulation culture was incubated with 15 μL of 2 mg/mL Zymolyase (Seikagaku Corporation) for 30 min at 30° to digest the spore ascus wall. Treated tetrads were dissected to individual spores using a micromanipulator microscope (Singer Instruments, Somerset, UK) on YEPD plates.

### PCR procedure

As DNA template, either 5 μL of cells suspended in water or 50 ng of purified DNA (DNA YeaStar Kit, Zymo Research) were used in a total reaction volume of 20 μL. The PCR mix also contained: 1.66 μM of each forward and reverse primers, 2.5 mM MgCl_2_ (Applied Biosystems), 0.66 mM dNTPs mix (Larova), 2 μL buffer (Applied Biosystems), 0.5 μL Taq DNA polymerase (1.5 units), and PCR grade water to complete the volume. The PCR profile used for amplification was 94° for 10 min, followed by a touchdown profile: 94° for 30 s, annealing from the upper to the lower temperature for 1 min with a decrease of 0.5° per each of 14 cycles, and extension at 72° for 2 min. The touchdown cycles were followed by 23 cycles with the lower annealing temperature and a final elongation step in 72° for 10 min. PCR products were verified by electrophoresis on 1.5% TBE Agarose gel (Lonza) containing Ethidium Bromide (Apex BioResearch).

### Genotyping

Strains' genotype was determined either by SNP HRM analysis with fluorescent DNA intercalating dye, or by sequencing using big dye chemistry on an ABI PRISM 3730XL DNA Analyzer at the Center for Genomic Technologies, Hebrew University, Jerusalem, as described in Shapira et al. (2014). Primers used for both techniques are detailed in Supplementary Table 1.

### Transformation and allele replacement

Transformations of linear DNA fragments into yeast genome were performed following standard LiAc method (Gietz and Woods, 2002). Gene knockout was done by replacing the sequence of interest by the *MX4* cassette containing a resistance gene to either G418 (*KanMX4*) or Hygromycin B (*HygMX4*; Vorachek-Warren and McCusker, 2004). Replacing the native allele of a gene with an allele from another strain was done by the two-steps transformation method (Storici et al., 2003). In the first step, by replacing the gene of interest with a cassette containing drug resistance and the *URA3* gene homolog and in the second, by replacing the cassette with the alternative allele and selecting successful replacements on 5-FOA SC plates. Successful integration into the genome was confirmed by lack of growth on uracil-lacking SC plates and by PCR using position specific primers. The primers used in transformation procedures are detailed in Supplementary Table 1.

### Yeast populations

#### F1 population

In Shapira et al. (2014), forty-four tetrads of the hybrid between S1001 (isogenic to S288c) and SK1 strains were dissected to isolate 176 F1 haploid spores. In this study, these segregants were genotyped by HRM at five different genes to determine which parental allele each segregant had in each gene. This genotypic information allowed assigning genotypes to the diploid segregants of the following populations.

#### Backcross populations

In Shapira et al. (2014), two populations of reciprocal backcrosses were constructed by backcrossing each of the haploid F1 spores twice, once to S1001 haploid to generate the BC1-S1001 population, and once to SK1 haploid to generate the BC1-SK1 population. The doubling time (DT) values of the diploid segregants from our previous study were used in conjunction with the genotypic values obtained in this study for the analyses. The DT data is available from Shapira et al. (2014).

#### F2 population

A hybrid strain between S1001 and SK1 containing HygB and G418 resistances in its *HO* locus was sporulated and 24 of its tetrads were dissected to obtain 96 haploid spores. The mating type, G418 or HygB resistance and *AEP3* allele were determined for each spore. Sixteen *MATa*, HygB^+^ spores were crossed in all pair wise combinations to 24 *MAT*α, Kan^+^ spores to create a population of 350 F2 diploids. Since the *AEP3, CRS2*, and *RRP3* alleles of each haploid were determined, the crosses created three genotypic groups for each gene: homozygous for S1001 allele, homozygous for SK1 allele, and heterozygous. Each group contained several tens of strains according to the distribution of alleles in the chosen F1 haploid parents. For ADR1 and Win. 828, only two genotypic groups were available, homozygous for SK1 allele and heterozygous because of the way the crosses of F1 haploids were done. Selection of diploids was done on double drug plates and verified by *MAT* locus PCR.

## Growth phenotype analyses

The phenotypic and genotypic data of all strains measured in this study were stored in datasets according to the different experiments and these are given as Supplementary Data Sheet 2. Any experiment that measured growth of strains on more than one 96-wells plate, included the same reference strain on each plate. The average of that strain was used to estimate plate effect and normalize the growth of other strains allowing strain comparisons across plates and experiments. All statistical analyses were done using the statistical software JMP 8 (SAS Institute).

## Analysis of allele replacement strains

Haploid strains from SK1 and S1001 genetic backgrounds were used in the two-steps construction of allele replacement strains (see above for details on the procedure). In the first step, the original allele is deleted and replaced by a cassette containing a drug marker and in the second step the cassette is replaced with the introduced allele. For the analysis of haploid strains, gene-deleted strains from the first step and allele-replaced strains from the second step were used. For the analysis of diploids, nine genotypic groups were produced using the original and allele-replaced strains of both backgrounds. The nine groups were: three *AEP3* genotypic groups, homozygotes for S1001 allele, homozygotes for SK1 allele and heterozygotes in each of the SK1, S1001, and hybrid backgrounds. DT-values of all strains (haploids and diploids) were measured at 30° and 37° using multimode readers and the mean DT-values of two technical repeats were calculated and used for the statistical analyses.

## Heterosis measurements

Doubling time (DT)-values of strains were measured and calculated as detailed in our previous work (Shapira et al., 2014). For statistical analyses that compared growth between strains, Log_2_DT-values were used. In order to identify loci contributing to heterosis, the heterotic phenotype of hybrids was calculated based on DT-values of the hybrid and its parents in YEPD 37°. Heterotic value of each hybrid was calculated according to the following categories:

If the hybrid (*Hy*) was faster than its faster parent (*FP*), its heterotic value was calculated as:

$$\begin{array}{l} \left(FP-Hy\right)∕FP \end{array}$$

If the hybrid was slower than its slower parent (*SP*), its heterotic value was calculated as:

$$\begin{array}{l} \left(SP-Hy\right)∕SP \end{array}$$

If the hybrid DT-value was within the range of the parents, its heterotic value was set to zero. Heterotic values of the 120 hybrids were used in the genome-wide scan to identify candidate over-dominant loci. Zeroing the value of all the hybrids that were within the range of the parental lines, left for the following scan only the phenotypic variability of those hybrids that exceeded the parental range.

Another measure for level of heterosis, the degree of dominance (*d*/*a*), was calculated for each hybrid and furthermore for each F2 genotypic groups, by using the following parameters: Mid-parent value (*m*)—Mean of homozygous parents phenotype:

$$\begin{array}{l} \text{Mid-parent value}:m=\left[\frac{\left(P1+P2\right)}{2}\right] \end{array}$$

Additive genetic deviation (*a*)—The difference between each of the homozygous parents and the mid-parent value:

$$\begin{array}{l} \text{Additive genetic deviation}:\;a=\left(P1\;or\;P2-m\right) \end{array}$$

Dominant genetic deviation (*d*)— deviation of the heterozygous hybrid (*Hy*) from its mid-parent value:

$$\begin{array}{l} \text{Dominant genetic deviation}:\;d=\left(Hy-m\right)\\ \text{Degree of dominance }\left(d/a\right):\frac{d}{a}=\frac{\left[Hy\;-\;\frac{\left(P1+P2\right)}{2}\right]}{\left[P1\;or\;P2\;-\;\frac{P1+P2}{2}\right]} \end{array}$$

Degree of dominance values were used in the analysis of the hybrid-specific background loci, in the analyses of populations (RBC1 and F2), and in the analyses of deletion and allele-replacement strains.

## Genome-wide scan for loci with an over-dominant effect

The parental strains used for making hybrids were fully sequenced and the list of SNPs for each strain that was called in that study (Liti et al., 2009) was used here. This list of polymorphism included only SNP genotypes and no other types of polymorphisms. Here, based on these SNP genotypes, a computational method that associated mode of inheritance (heterozygosity in SNPs) with phenotype (level of heterosis at 37°) was developed for yeast following a similar method applied to plants (Ben-Israel et al., 2012). Briefly, a sliding window algorithm was used to divide the SNP data of the 16 parental strains to genomic windows containing between two and nine haplotypes, with three to ten SNPs in each. The genotype of hybrids was inferred from their known parental genotypes. For each genomic window, the 120 hybrids were divided into genotypic groups: homozygotes for any of the haplotypes in the window and heterozygotes for any combination of haplotypes. An example of such grouping in a window that contained two haplotypes and thus, three genotypic groups is given in Supplementary Figure 1A. In windows containing more than two haplotypes, only haplotypes found in three or more parents were considered for analyzing the phenotypic differences between local homozygotes and heterozygotes. The means of the heterotic values, calculated as described above, of three genotypic groups (two homozygous and the corresponding heterozygous) were compared (Kolmogorov–Smirnov test, *P* < 0.05). In every window with a significant difference, the three genotypic groups that yielded the most significant difference were chosen. Genomic windows in which the mean heterotic value of the heterozygous hybrids group was significantly larger than the means of both homozygous groups were considered as candidate loci with an over-dominant effect. Finally, since multiple comparisons were carried out, to reduce the chances for false positives identification, we applied a Benjamini–Hochberg correction at an FDR level of 0.1 to the list of significant loci.

## Identification of candidates in a specific hybrid-background

The set of hybrids in the heterozygous group varied among candidate windows. From the list of significant windows prior to the FDR correction, we chose 74 windows for which the specific SK1xS1001 hybrid was included in the group of heterozygote hybrids. This was a logical filtering step since over-dominant contribution is expected only from heterozygous loci. We then applied to these 74 windows a more stringent analysis to identify the more promising over-dominant loci in this hybrid background. For each window, we downloaded sequence alignments of the 16 parental strains from the SGRP blast server (http://www.moseslab.csb.utoronto.ca/sgrp/blast_original/). Parental haplotypes were deduced from the sequences and hybrids were reassigned to three genotypic groups in each window based on the haplotype combination of their parents. Due to the differences between the SNP list and the full sequence alignments, changing a haplotype call for even one parent per window, changed the genotypic grouping of the hybrids and could have changed the results of the statistical tests. In practice, grouping of hybrids in all 74 windows changed in the second analysis compared to the first scan. Therefore, using all sequence polymorphisms, not only SNPs, made this second analysis more stringent. Furthermore, instead of assigning heterotic values to hybrids as in the initial screen, in this second analysis we assigned them with continuous *d*/*a*-values. The heterotic value was set to zero in the initial scan for about half of the hybrids that grew within the parental range while *d*/*a*-values in the analysis of the 74 candidates were assigned to all hybrids. Zeroing values for half of the hybrids gave more weight to the other half (the heterotic hybrids) and favored the identification of over-dominant loci in the initial analysis. On the other hand, including continuous *d*/*a*-values for all hybrids in the second analysis, increased the variance around the mean for each genotypic group (Hom1, Het, and Hom2) and this larger variance made it harder to reach statistical significance. An example of the spread around the mean of each genotypic group is given in Supplementary Figure 1B. Using continuous *d*/*a*-values for all hybrids is the second reason why the second analysis was more stringent.

Based on the sequence alignments, from the list of 74 loci, 12 were filtered out since either SK1 and S1001 shared the same haplotype, or the new haplotypes assignment created parental groups with less than three parents. The 62 remaining loci were reanalyzed to compare the three genotypic groups of the hybrids using both Kruskal–Wallis aparametric test and the more stringent Tukey–Kramer HSD parametric test. We applied a Benjamini–Hochberg correction at an FDR level of 0.1 to the list of significant loci. All statistical analyses were done using the statistical software JMP 8 (SAS institute).

## Analysis of dominant and epistatic effects in the F2 population

The F2 population was used to evaluate the mode of inheritance of candidate loci. First, we compared the mean DT between the three genotypic groups (Hom1, Het, and Hom2) for each locus using a Tukey–Kramer HSD test. Secondly, we determined the mode of inheritance by comparing *a*- and *d*-values in each locus. The additive genetic deviation (*a*) was half the difference between means of the two homozygotes and the dominant genetic deviation (*d*) was the difference between the mid-parent and the mean of the heterozygous segregants. Positive *d*-values were assigned when the mean growth of heterozygotes was faster than the mid-parent whereas negative *d*-values were assigned when slower. Larger *d*- than *a*-values indicate over-dominance. To compare between *a*- and *d*-values, each F2 diploid was assigned with either *a*- or *d*-values (its difference from the overall mid-parent value) if it was a homozygote or a heterozygote, respectively. We compared the mean *a*- and *d*-values for each locus using a *t*-test. For the analysis of epistasis between pairs of candidate loci, we compared the *d*-values of a given locus with and without heterozygotes in another locus using a *t*-test.

## Results

### Associating loci with non-additive mode of inheritance with growth

We sought to identify loci that show a non-additive mode-of-inheritance and therefore, have a non-additive effect on the phenotype and not just an effect due to substitution of alleles. We associated our previous phenotypic measurements of hybrid growth at 37° (Shapira et al., 2014) with the SNP data of the 16 parental strains available from the SGRP collection (https://www.sanger.ac.uk/research/projects/genomeinformatics/sgrp.html). Each SNPs-containing genomic window defined a set of haplotypes for the haploid parental strains and based on the haplotypes of their parents, the 120 hybrids were categorized as heterozygotes or homozygotes to one or the other haplotypes (Supplementary Figure 1A). The mean level of heterosis at 37° was compared between homozygous and heterozygous hybrid groups in each window to identify loci in which heterozygotes significantly outperformed homozygotes (Supplementary Figure 1B). Altogether, 50,318 windows were tested. The distribution of haplotype number per window ranged between two and twelve with a median of four haplotypes (Supplementary Figure 2A). Haplotypes contained three to ten SNPs per window with a median of three (Supplementary Figure 2B). The window size ranged from 2 to 11,289 bp with a mean of 153 bp and a median of 97 bp (Supplementary Figure 2C). These statistics reflect on the variability in polymorphism levels and on the extent of haplotype blocks in different regions of the genome. In accordance with the gene content of the yeast genome, about 70% of the windows were inside annotated ORFs and based on the analysis parameters, a single ORF included 1–82 different windows.

**Figure 1:**
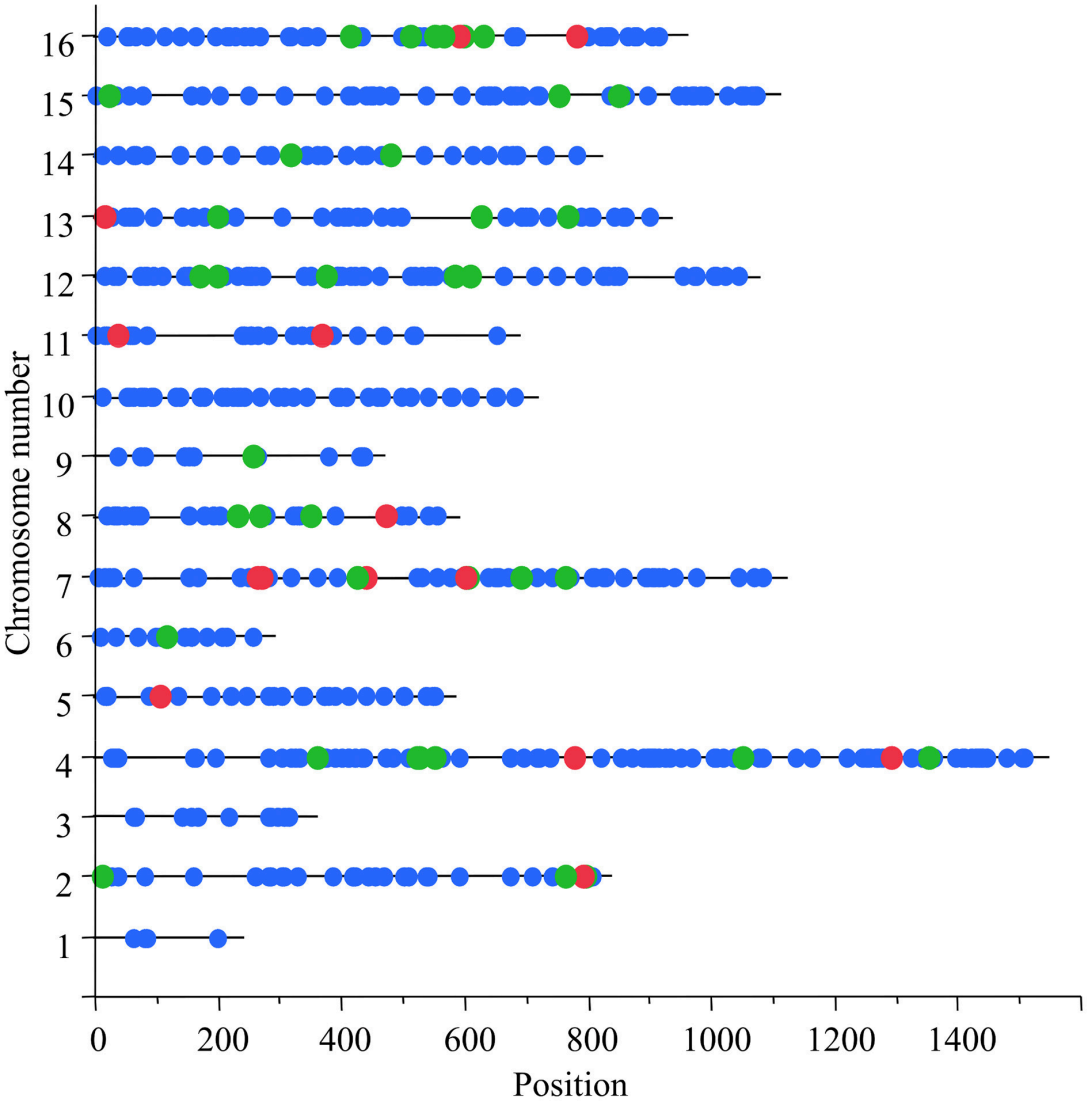
**Genomic distribution of candidate over-dominant loci**. X-axis denotes the position along the chromosome (in Kb) and Y-axis denotes the chromosome numbers. Each dot marks one of the 803 windows that were found to be significant in the genome-wide scan. Green dots mark the 55 potential over-dominant windows in the SK1xS1001 background and red dots the 12 statistically significant ones.

The size of a window was set computationally such that each haplotype in it was found in at least three parental strains. Therefore, the small median size and the small SNP number inside each window indicated high level of strain divergence and short spans of linkage disequilibrium. In 803 out of 50,318 windows that were tested, the mean heterosis value of the local heterozygous hybrids was significantly different from the means of both local homozygous hybrid groups (Supplementary Data Sheet 1). These significant loci were divided into 792 windows with an over-dominant effect and 11 with an under-dominant effect. Both over and under-dominant windows were distributed across the chromosomes (Figure 1).

Since we carried out over 50,000 statistical comparisons, we applied to the list of 803 significant loci a Benjamini–Hochberg correction at an FDR level of 0.1. Following this correction, none of the 803 loci remained significant at α = 0.05 level. Therefore, this list of loci is suggestive and probably includes false positives. However, we note that what this statistical correction does is ruling out loci with lower significance and these correspond not only to false positive but also to minor effect loci. Furthermore, the significance of a test is also contingent upon the variance around the mean of each group. The variance around the means of the three genotypic groups (Hom1, Het, and Hom2) in our tests was on average over 90% of the total variance (see Supplementary Figure 1B for an example). Therefore, even the loci with the largest effect explained only a small fraction of the total variation. Notwithstanding the possibility that the list of 803 loci includes false positives, we continued in the analyses considering that some loci would be true positives with minor effects. The latter option is intriguing since it implies that heterosis can be gained by accumulation of contributions from many loci, each with a minor effect. Furthermore, in yeast, testing experimentally the contribution of loci, even if they have a minor effect, is more readily available. Therefore, we continued analyzing the list of 803 loci in order to identify in it suitable candidates for further experimental testing.

### Candidate loci in a specific hybrid background

The computational screen identified candidate loci based on comparisons among haplotypes of 120 hybrids, representing allelic variation in all 16 parental lines. In order to test experimentally the contribution of a given candidate locus, the list was narrowed down to loci that were heterozygous in a specific hybrid background. From the hybrids that in the analysis of Shapira et al. (2014) showed heterotic growth at 37°, the hybrid between S1001 and SK1 strains was chosen for further analyses. In the SK1xS1001 background, 74 of the 803 listed loci were heterozygous and these were spread across the whole genome.

To choose loci for further analysis from the list of 74 background specific candidates, we first reanalyzed all 74 using a more stringent analysis. In this second analysis, to determine the parental haplotypes, we used the full genomic sequence of each window rather than just SNP genotypes and we used the degree of dominance (*d*/*a*) as the phenotypic value of hybrids rather than their heterotic value (see Methods section for details). Based on the full sequence alignments, haplotype assignment changed for at least one of the 16 parental strains in each of the 74 windows compared to the assignment made based on the SNP genotypes alone. The changes in parental haplotype assignments were first because polymorphisms other than SNPs (mainly indels) were included, and in addition because the SNP content in the window changed (due to possible sequence updates or because SNP were accepted to or rejected from the list based on the SNP calling algorithm settings). Consequently, grouping of hybrids into the three genotypic groups (Hom1, Het, and Hom2) also changed. Hence, we compared the means of the updated three genotypic groups for the 74 windows using *d*/*a* as the measure for heterosis. We note that it would have been technically very challenging to carry out the genome-wide scan in the same way as the manual analysis of the 74 windows was done.

Due to the new haplotype assignments, 12 windows were filtered out since either the same haplotype was shared by SK1 and S1001, or there were less than three parental strains in one of the haplotype groups (see Criteria for Analysis in the Methods Section). Based on the comparison among means, for 53 of the 62 analyzed windows, the local heterozygous group had a tendency to grow faster than the local homozygous groups (tendency for over-dominance), and for 2 of the 62, the heterozygous group had a tendency to grow slower than the homozygous (tendency for under-dominance). In total, 55 of the 62 windows (89%) showed the expected trend among the means. For 12 of the 55 (22%), the differences were significant (*P* < 0.05) based on a Kruskal–Wallis non-parametric test and after correcting for multiple testing at an FDR level of 0.1. These significant loci were scattered across the genome (Figure 1).

**Figure 2:**
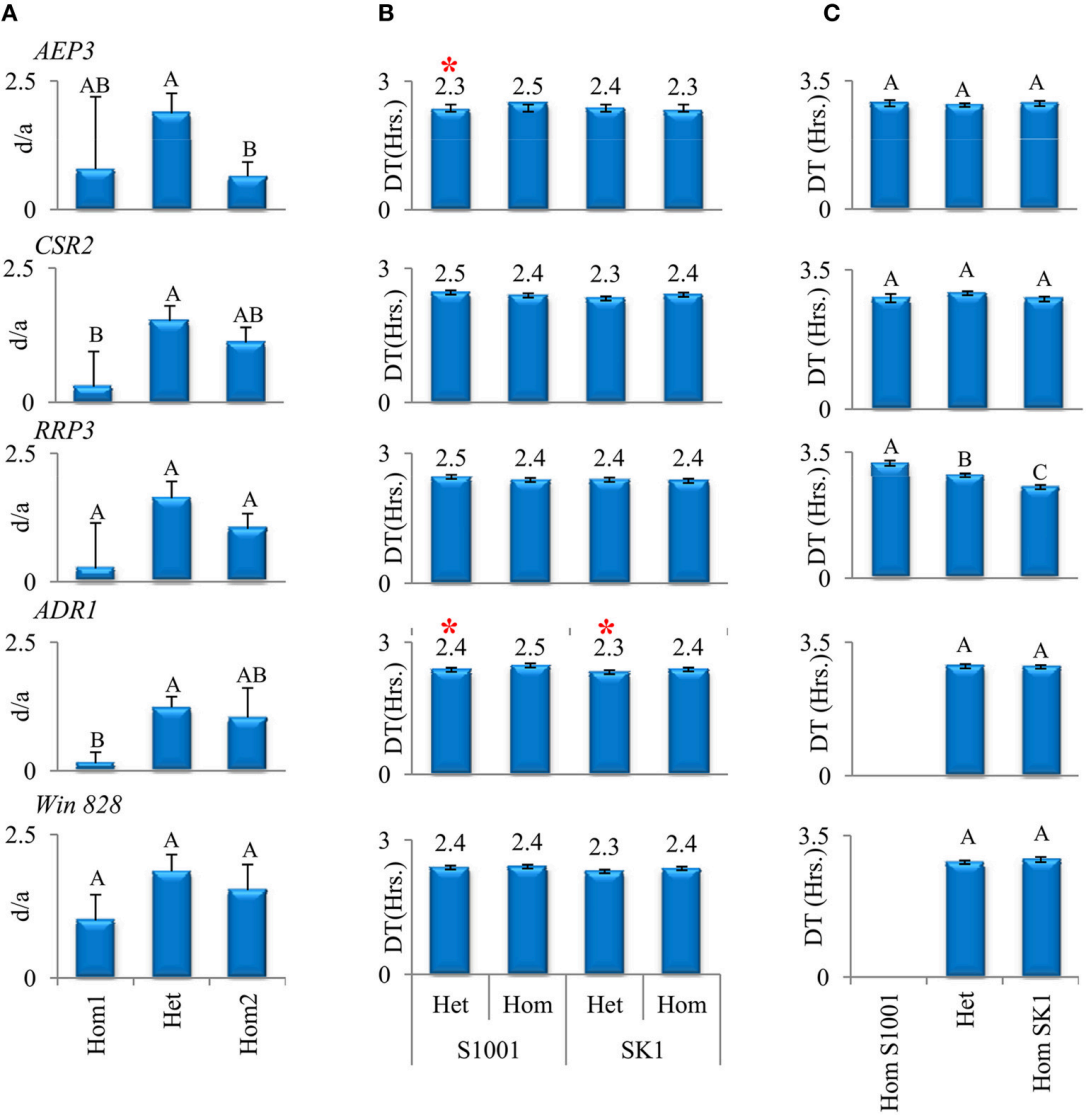
**Effects of five candidate loci in three analyses**. **(A)** Reanalysis of 120 hybrids in three genotypic groups. Mean d/a (+/−StdErr) for three genotypic groups of hybrids (heterozygotes and homozygotes for one or the other allele) were compared using Tukey–Kramer HSD test. Different means in the TK test are denoted by different letters. **(B)** Analysis in RBC1 populations. Mean DT (+/−StdErr) at 37° for each window. Hom and Het denote homozygotes and heterozygotes for the allele, respectively. Significant differences between local heterozygotes and local homozygotes are marked by asterisks (Wilcoxon test, *P* < 0.05). **(C)** Analysis in F2 population. Mean DT (+/−StdErr) at 37° of each local genotype for each window. Different means in the TK test are denoted by different letters.

Therefore, despite the non-trivial move from the initial list of 803 putative loci to a background specific list of 12 significant candidates, the results supported our initial view. Even for the most significant loci, the differences among the means of the three genotypic groups (Hom1, Het, and Hom2), which represent the effect of each locus, explained less than 10% of the total variance among hybrids (Figure 2A). Therefore, much like in the collection of 120 hybrids, also in a specific hybrid background there were several loci, each with a relatively minor effect, which contributed to variation in heterosis among hybrids.

### Testing effects of candidate loci in reciprocal backcross populations

Based on the second, more conservative, analysis that identified significant candidates in the SK1xS1001 hybrid background, five windows were selected for further analyses based on the consistency of test results between the initial scan and the background-specific analysis. These five windows showed the expected heterotic relationship, i.e., the heterozygotes group had a marked tendency to grow faster than the homozygotes groups (Figure 2A). Four windows were inside annotated genes: *AEP3* (Chr. XVI), *CSR2* (Chr. XVI), *RRP3* (Chr. VIII), *ADR1* (Chr. IV) and one, window # 828, was located in an intergenic region (Chr.XII, coordinates 198411–198485). Due to large variation in *d*/*a*-values among different hybrids within each genotypic group, significant differences between the groups were found in three of the five loci, based on the more stringent Tukey–Kramer parametric test.

The first analysis of the five candidate loci was in segregating progeny. In Shapira et al. (2014), we used a population of 176 haploid F1 segregants from the SK1xS1001 hybrid to construct two reciprocal backcross 1 (RBC1) populations by crossing the F1 segregants to each of the SK1 (BC1-SK1) and S1001 (BC1-S1001) parents. In our previous study, doubling time (DT)-values of haploid F1 segregants and two diploid RBC1 populations were measured at 37° and as a control, at 30°. The mean DT of BC1-SK1 was smaller than that of BC1-S1001, indicating that the SK1 genetic background contains more beneficial growth alleles. Phenotypic distributions at both temperatures exposed the quantitative nature of growth rate and attested to the segregation of the alleles affecting these traits (Supplementary Figure 3).

For this study, high-resolution melt (HRM) genotyping assay was developed and used to determine which F1 segregant carried what parental allele in each candidate locus. The genotype of each diploid BC1 segregant (homozygous or heterozygous) in each candidate locus was deduced based on the combined genotype of the specific F1 and the common backcross parental strain. In this analysis, a comparison was made between means of two groups, either homozygous or heterozygous in the candidate locus, but otherwise with segregation of alleles in the rest of the genome. Thus, this comparison tested the contribution of a specific candidate locus, while averaging out the contribution of other loci. For half of the comparisons, the local heterozygotes showed a tendency to grow faster than the local homozygotes. For *AEP3* and *ADR1*, local heterozygotes grew significantly faster than local homozygotes (Figure 2B). *AEP3* is a gene that encodes for a protein that may facilitate use of unformylated tRNA-Met in mitochondrial translation initiation (Ellis et al., 2004; Lee et al., 2009), and *ADR1*, a gene that encodes a carbon source responsive transcription factor (Young et al., 2003). Both genes were not associated before with growth at high temperature, let alone heterosis in growth.

### Testing effects of candidate loci in an F2 population

Since in each RBC1 population the local heterozygote could be compared with one parental homozygote at the time, we crossed several haploid F1 segregants and created an F2 population of 350 diploids. Genotype of the diploid F2 segregants in each of the five candidate loci was determined according to the combination of F1 parental alleles. In the F2 population, three genotypic groups were available for *AEP3, CSR2,* and *RRP3,* whereas for *ADR1* and *Win. 828*, homozygotes for the SK1 allele and heterozygotes were available. Doubling time values of F2 segregants at 37° (Supplementary Data Sheet 2) were variable with a mean of 2.84 and a standard error of 0.034. In the comparison among the mean DT of the genotypic groups, a significant difference was found for *RRP3* and the relationship among genotypes indicated a co-dominance mode of inheritance. In all other four loci, only tendencies could be observed. Consistent with previous analyses, for *AEP3*, a tendency for over-dominance was found (Figure 2C). For *CSR2*, a tendency for under-dominance was found, whereas for *ADR1* and *Win. 828*, SK1 homozygotes grew slightly faster and slower, respectively, than heterozygotes (Figure 2C).

To look more carefully on the mode of inheritance for *AEP3, CSR2,* and *RRP3*, we calculated the additive (*a*) and dominant (*d*) genetic deviations as well as the degree of dominance (*d*/*a*) based on the F2 phenotypes (Table 1). For *AEP3*, the dominant deviation was positive and significantly larger than the additive deviation, indicating that *AEP3* had a minor yet significant over-dominant effect. For *CSR2, d* was negative and significantly smaller than a, indicating an under-dominant mode of inheritance. For *RRP3, d* on average equaled zero and was significantly smaller than *a*, indicating a co-dominant mode of inheritance. The *a* and *d* comparisons augmented the trends observed in the DT analysis with statistical significance. Overall, these two F2 analyses supported the contribution of *AEP3* to heterosis. For the other four loci, different results were obtained compared to the association scan and RBC1 analysis.

**Table 1:** **Additive (a) and dominant (d) genetic deviations in the inheritance of *AEP3, CSR2,* and *RRP3* in the presence or absence of heterozygotes for each of the other four loci**.

	***AEP3***			***CSR2***			***RRP3***		
	***a***	***d***	***d/a***	***a***	***d***	***d/a***	***a***	***d***	***d/a***
Including all F2	0.01	0.06^*^	4.48	0.01	−0.10^*^	7.63	0.32	0.00^*^	0.01
**EXCLUDING HET GROUP OF**									
*AEP3*				0.05	−0.12	2.30	0.24	0.11^**^	0.45
*CSR2*	0.13	0.04	0.31				0.20	0.05	0.24
*RRP3*	0.12	0.10	0.80	0.19	−0.23	1.21			
*Win. 828*	0.03	−0.21**	8.22	0.24	−0.13	0.54	0.25	−0.23^**^	0.92
*ADR1*	0.04	−0.11^**^	3.05	0.04	−0.10	2.74	0.37	−0.03	0.09

Negative d-values stand for slower growth of the heterozygote group relative to the mid-parent value.

* The dominant deviation d was significantly different from the additive deviation a (Wilcoxon rank test, P < 0.05).

** Significant change (Wilcoxon rank test, P < 0.05) compared to the value based on including all F2 segregants.

### Testing epistatic effects among loci in the F2 population

The heterotic effect of mainly *CSR2* and *RRP3* was not consistently significant in the association scan, RBC1, and F2 analyses. This could happen because the individual effect of each candidate is minor and therefore significant in one analysis but not in another. However, it could also happen because the heterotic effect of one locus is contingent upon effects of other loci. Using the phenotypic and genotypic data of the F2 population, we could test if there were epistatic relationships between pairs of candidate loci. We used a stratification strategy, by which the heterotic effect for each of the *AEP3, CSR2,* and *RRP3* genes were re-evaluated after excluding the heterozygous F2 segregants in each of the other candidate genes. Since the heterozygous genotype makes the heterotic contribution, excluding the heterozygous F2 segregants from the analysis of one locus will cancel its heterotic contribution and allow evaluating changes in the contribution of other loci. For example, comparing d of the *AEP3* locus with and without the F2 *CSR2* heterozygotes allowed inferring whether an epistatic relationship between these two loci existed (Table 1).

Using this method, we found that in the absence of *Win. 828* and *ADR1* heterozygotes, *AEP3* effect was reversed and became under-dominant (*T*-test, *P* < 0.05). Furthermore, *CSR2* effect was not affected by heterozygotes of other loci although both *AEP3* and *CSR2* locate to chr. XVI and the estimated genetic distance between them is 17 cM based on the recombination events counted in SK1xS1001 F1 segregants (Wilkening et al., 2013). Lastly, the effect of *RRP3* decreased from co-dominant to fully-recessive in the absence of *Win. 828* heterozygotes. These changes in *d* were not accompanied by significant changes in a, indicating that indeed we analyzed effects that were brought about by heterozygosity. Thus, using the stratification method, epistatic relationships between loci were identified that contributed to the overall effect of these loci. Since in the presence of heterozygosity in combinations like *AEP3-Win. 828, AEP3-ADR1,* and *RRP3-Win. 828,* the *d*-value of *AEP3* or *RRP3* were larger compared to in its absence, the epistatic relationships we found between these pairs were synergistic.

### *AEP3* effect in haploid strains

**Figure 3:**
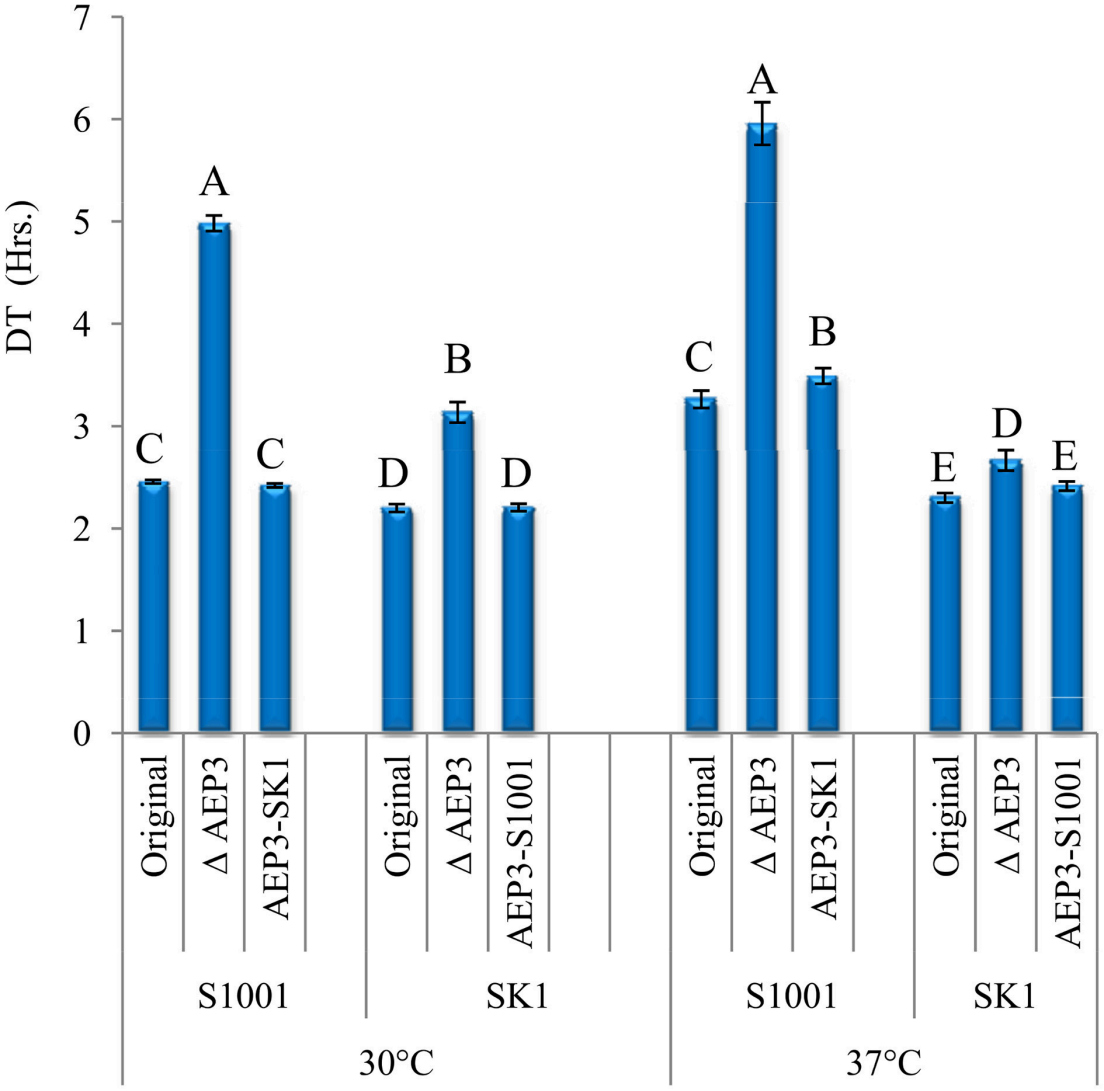
**Effect of *AEP3* allele replacement in haploid strains**. Comparison of DT-values at 30° and 37° of *AEP3* genotypes. At each temperature, three haploid strains in each genetic background were compared: original haploid, original after *AEP3* deletion and the original after introducing the corresponding *AEP3* allele from the other strain. Mean Log_2_DT was compared among six strains at each temperature by a Tukey–Kramer HSD test. Bars with different letters denote genotypes with different growth rates, separately for each temperature (*P* < 0.05).

Given the previous findings that supported more consistently the heterotic effect of *AEP3*, we further focused on its contribution to heterosis at 37° by allele-replacement experiments. The allele of each parental strain was replaced with the corresponding allele from the other parent in haploid strains. Since allele deletion was the first stage in constructing allele-replacement strains, we included also the gene-deleted haploids in this comparison (Figure 3). DT-values comparison indicated that *AEP3* deletion slowed down significantly the growth in both parental backgrounds, at both temperatures, verifying the contribution of this gene to growth. At 30°, both alleles contributed equally to growth in both genetic backgrounds. Nevertheless, at 37° introducing the SK1 allele into the S1001 haploid slowed down growth compared to the original S1001 haploid. In the SK1 background, the S1001 and SK1 alleles had a similar effect on growth at 37°. Consistently with the epistatic effects found in the F2 population, gene-deletion analysis indicated that the effect of the *AEP3* allele on growth at 37° depended on genetic background.

### *AEP3* effect in diploid strains

**Figure 4:**
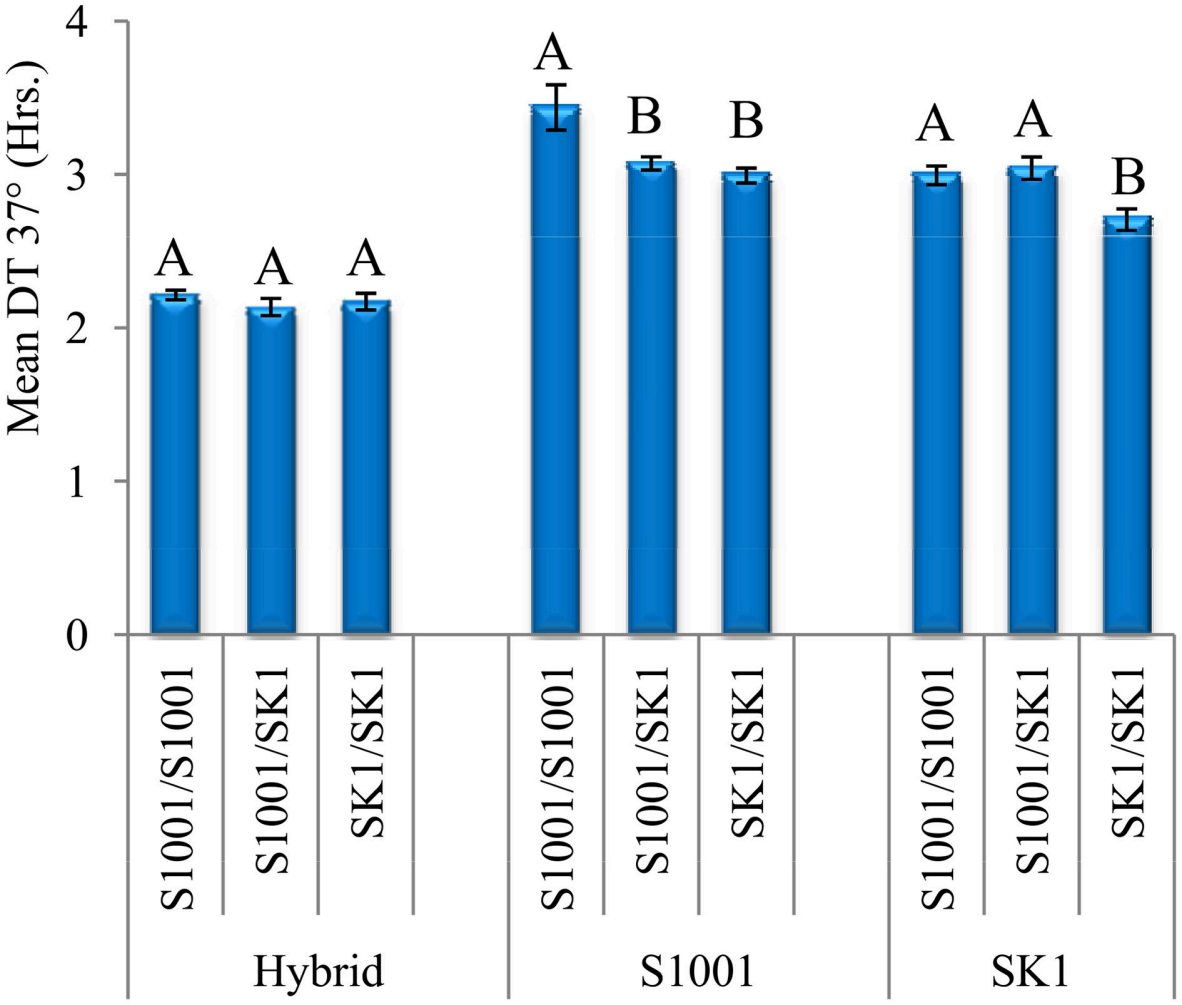
**Effects of *AEP3* genotypes in three diploid genetic backgrounds at 37°**. Comparisons of DT between local genotypes of *AEP3* (homozygotes to S1001 allele, homozygotes to SK1 allele and heterozygotes) were carried out in three diploid genetic backgrounds: heterozygous SK1xS1001 hybrid, S1001 homozygous and SK1 homozygous. Within each genetic background, the differences between three *AEP3* genotypes were tested by a Tukey–Kramer HSD test. Bars show means and StdErr of DT at 37°. Different letters denote a significant difference (*P* < 0.05), separately for each genetic background.

Testing gene contribution not only to growth but to heterosis is better if carried out in diploids. *AEP3* allele-replacement strains were constructed in three diploid backgrounds: heterozygous hybrid and both homozygous parental backgrounds. Differences among Log_2_DT-values of the strains were analyzed by a two-way ANOVA model with *AEP3* genotype (S1001 homozygote, SK1 homozygote, and heterozygote) as one main factor and genetic background (hybrid, S1001, and SK1) as another main factor (Table 2). A significant interaction between the main factors was found for both temperatures indicating again that the effect of *AEP3* genotype varied depending on the genetic background. Furthermore, regardless of the interaction, this experiment demonstrates well the general heterosis of the hybrid at 37°, since regardless of the *AEP3* genotype, all hybrid background strains grew faster than the strains of the two parental backgrounds (Table 2 and Figure 4).

**Table 2:** **Mean DT and StdErr-values of three *AEP3* genotypes in three genetic backgrounds at 30° and 37°**.

		**30**^**°**^			**37**^**°**^		
		**S1001/S1001**	**S1001/SK1**	**SK1/SK1**	**S1001/S1001**	**S1001/SK1**	**SK1/SK1**
Hybrid	Mean	1.78^de^	1.72^e^	1.84^cde^	2.21^d^	2.14^d^	2.17^d^
	(StdErr)	(0.01)	(0.01)	(0.06)	(0.03)	(0.06)	(0.05)
S1001	Mean	2.36^a^	2.10^b^	2.12^ab^	3.44^a^	3.07^ab^	2.99^b^
	(StdErr)	(0.14)	(0.02)	(0.02)	(0.15)	(0.04)	(0.05)
SK1	Mean	2.00^bc^	2.06^b^	1.96^bcd^	2.99^bc^	3.04^b^	2.71^c^
	(StdErr)	(0.02)	(0.05)	(0.01)	(0.06)	(0.07)	(0.07)

In rows are the genetic backgrounds and in columns the allelic composition in the AEP3 gene. Different letters in each temperature mark significant differences (Tukey–Kramer HSD test, P < 0.05).

We tested the differences between *AEP3* genotypes separately in each genetic background at 37° due to the significant *AEP3* genotype by genetic background interaction (Figure 4). In the hybrid background, the local heterozygote genotype grew on average faster than both homozygotes. This effect of *AEP3* was significant by an ANOVA test but not by the more stringent Tukey–Kramer HSD test. In both S1001 and SK1 homozygous backgrounds, SK1 allele in *AEP3* improved growth, but in a background specific manner. In the S1001 background, having one or two *AEP3-*SK1 alleles improved growth to a similar extent, demonstrating a fully dominant effect in the direction of improving growth. In the SK1 background, having one *AEP3-*SK1 allele did not improve growth but having two did, indicating that the SK1 allele had a fully recessive effect in the direction of improving growth (Figure 4). Taken together, the trend for an over-dominant effect of *AEP3* in diploids supported its contribution to heterosis at 37° in the hybrid background. Significant dominant effects but in opposite directions were observed also in the parental backgrounds, confirming the dependence of the *AEP3* effect on heterozygosity in other loci that was found by the F2 population analyses.

## Discussion

Many traits of interest in humans, crop plants and farm animals have a complex genetic basis. Typically, QTL identification relies on finding allelic variation that consistently co-segregates with phenotypic differences (Mauricio, 2001; Altshuler et al., 2008; Frazer et al., 2009; Zhang et al., 2012; Solberg Woods, 2014). Heterosis can also be considered as a quantitative trait (East, 1936; Birchler et al., 2010) as it has various degrees, but to identify loci affecting heterosis, genotypic states, and not only allelic variation should co-segregate with phenotypic differences. Therefore, past heterosis studies in crop plants used sets of backcrosses (a strategy we employed in the second stage) and crosses of advanced recombinant inbred lines to link between heterozygosity and trait differences (Hua et al., 2003; Lariepe et al., 2012). Crosses containing segregating progeny have the main advantage of testing loci effects in the background of a mixed genome. However, producing backcrosses, F2 and advanced inbred lines for several genetic background is a demanding task. Due to technological developments, DNA sequence data and genetic polymorphism information is becoming available not only for a limited number of individuals but also for many individuals of a population and for several variants of the same species. For yeast, this data is already available, allowing us to test a method for using polymorphisms among different strains to associate heterozygosity effects with trait differences rather than to use backcrosses of a particular genetic background.

The phenotypic data from a collection of 120 yeast hybrids that was previously constructed by pairwise crosses between 16 parental strains (Shapira et al., 2014) was analyzed here by a computational method that was adopted from crop plants (Ben-Israel et al., 2012) and adjusted for yeast. Consistent with our previous study in which we reported that heterosis was common in this hybrid collection (Shapira et al., 2014), the association method we used here identified almost exclusively loci with an over-dominant effect and only a few loci with an under-dominant effect. In the budding yeast, large sequence divergence between parental strains (Liti et al., 2009) and high recombination rate (Mancera et al., 2011; Wilkening et al., 2013) created a genetic structure with relatively short haplotype blocks. In other organisms with more recent evolutionary divergence, like human and domesticated species, less genetic variation accumulated between variants and the genetic structure is composed of larger haplotype blocks (Wall and Pritchard, 2003). These genetic structure considerations should be taken into account when using this method in animals and plants as they significantly affect the power to identify heterotic loci. Under the genetic structure of our collection, half of the 120 hybrids were heterotic for growth at 37° and we identified 803 significant, relatively short, windows with potential over-dominant effect on the trait for further consideration. The large number of loci and their relatively low statistical significance indicated that their effect size was minor. Therefore, this list probably contains loci with true minor effects alongside some false positives. What we would have identified first, had they existed, would have been loci with major contribution to heterosis. We therefore suggest that there are no major effect loci contributing to variation in heterosis at 37° in this hybrid collection. Given the high level of genetic variation, the many significant loci identified is consistent with the prevalence of heterosis in this hybrid collection (Shapira et al., 2014) and consistent with the idea that accumulation of effects from many loci, each with a minor dominant effect, could lead to an overall significant phenotypic heterosis (Chen, 2010). Our results provide empirical support for this view on the genetics of heterosis and therefore, identification of loci affecting heterosis in yeast might be as challenging or even more than identification of mild and minor effect loci affecting other quantitative traits.

One important aspect that stems from the genetic structure of our yeast collection is the level of genetic and phenotypic variation among parental strains and even more among hybrids. For each of the 803 potential loci (including the 74 background-specific loci), we tested the statistical significance of differences among the genotypic groups (Hom1, Het, and Hom2) by comparing them to the differences among hybrids within each group. In this hybrid collection, the genetic and phenotypic variations are both large. Due to the divergence that was found even in short windows, the parental strains were assigned to three or more haplotypes and consequently, per window, not all hybrids were included in the genotypic groups that were tested. Furthermore, almost half of the total phenotypic variation was attributed to differences within each genotypic group. Therefore, the power to detect significant loci by the genome-wide approach heavily depends on the number of genetic backgrounds, their genetic divergence and the phenotypic variation that their hybridization creates. The statistical power of genome-wide analyses, ours included, depends heavily on the sample size for detecting minor effect loci. Our analysis was based on 120 hybrids and tested over 50,000 loci. We identified loci with minor effects and hence with lower significance that did not pass the FDR correction for multiple testing. Nevertheless, we confirmed the minor effects of few candidates by follow up analyses. Therefore, our list of candidates probably contained some true positives with minor effects together with some false positives. *Post-hoc*, given the budding yeast divergence level, inclusion of more parental strains would have strengthened the genome-wide analysis and perhaps enabled better sorting out of false positives. Thus, the number and divergence of parental lines are important considerations in applying this method to other species.

Another important aspect, which ought to be considered when this association method is applied to different organisms, stems again from the number and the genetic structure of the parental strains. In other studies, constructing segregating populations originating from two parental lines limited the studied variation to that between these lines and dictated the genetic background in which further verification and analysis of specific loci would be carried out. In our case, the 803 potential loci were associated with heterozygote advantage based on the variation among all participating strains and thus, to further study the effect of specific loci, a choice of a specific hybrid and its parental strains was required. From the analysis of multiple genetic backgrounds, it was difficult to predict what the effect of a specific locus on heterosis would be in a specific hybrid background. In terms of a specific background, we chose SK1xS1001 hybrid for further analysis due to its heterotic growth at 37°. In terms of loci, out of 803 candidate windows, the ones that were heterozygous in this specific background were those that we further considered. Aside from selecting a hybrid and its heterozygous loci, moving from identifying loci based on the multi-strain analysis to testing the effect of candidate loci in a specific hybrid background represented a challenging step in this association method. The challenges were several and our workflow demonstrates the way we took to overcome these challenges. In practice, filtering the list of 74 using higher stringency analyses combining full-sequence alignments, degree of dominance values, and further statistical tests, narrowed down the list and allowed us to select five candidates to follow up further.

Similar to studies in crop plants, we tested the effect of the five candidates in segregating populations. In general, we found support for contribution of two loci to heterosis based on comparisons between local heterozygotes and local homozygotes in BC1 populations. However, consistent with the findings of the multi-strain scan, also in the specific background, the effects were minor. Whereas for *AEP3* and *ADR1*, significant differences were found in the BC1 analyses, for the other three candidates, only trends were observed. Based on growth measurements in the F2 population, *AEP3* had a tendency for over-dominance while *CSR2* and *RRP3* had tendencies for under-dominance and co-dominance, respectively. For all three genes, the dominant deviation was significantly different from the additive deviation, confirming that the trends found based on growth measurements were not just trends but rather significant effects. *AEP3, CSR2,* and *RRP3* had a significant over-dominant, under-dominant, and co-dominant mode of inheritance, respectively. Furthermore, since *CSR2* and *RRP3* that were initially identified as having over-dominant effects had later been shown to have either an under-dominant or an additive effect, the F2 analysis was also the first significant indication for inter-loci interactions that could explain these changing effects. Finding that *a*- and *d*-values of these loci changed significantly after elimination of heterozygous groups in other loci confirmed the effects of intra-locus interactions.

Since *AEP3* had the most consistent over-dominant effect, we verified its contribution by functional analyses. Based on the deletion analysis in haploids, we found that *AEP3* clearly contributes to growth in both parental backgrounds. Unlike its minor heterotic effect, the simple effect of *AEP3* on growth in haploids was considerable. At 37°, the original haploid grew 1.83 and 1.16 times faster than the Δ*AEP3* haploid for S1001 and SK1 backgrounds, respectively. However, the effect of the different alleles was minor and statistically significant only in the S1001 background at 37°. In diploids, we found an over-dominant effect for *AEP3* in the hybrid background. In the parental backgrounds, we found that the SK1 allele had either a fully dominant or a fully recessive effect. Finding support for the over-dominant effect in the hybrid background is important because this is the background in which *AEP3* was identified in the first place. Furthermore, the minor contribution of this single locus supported the view that a combination of several loci underlies the heterotic phenotype in this background. Finding significant changes in the effect as a function of the genetic background was the second clear indication for epistasis.

*AEP3* turned out to be the most convincing heterosis gene, contributing to both growth and heterosis at high temperature. The protein aep3 is localized to the peripheral mitochondrial inner membrane. Its function may facilitate use of unformylated tRNA-Met in mitochondrial translation initiation and it is stabilizing the bicistronic ATP8-ATP6 mRNA transcribed from the mitochondrial genome (Ellis et al., 2004; Lee et al., 2009). The S1001 and SK1 alleles of *AEP3* differ by seven SNPs. Three SNPs are synonymous and four are non-synonymous and create amino acid substitutions. The region upstream to the *AEP3* ORF, which might be considered as its promoter, differ by one deletion of 7 bp and one SNP, however, these polymorphisms does not overlap with currently annotated positions of features such as transcription factor binding sites. Therefore, comparing the sequences of these two alleles is not enough for identifying causative variants and further testing of specific substitution is required for that. The contribution of *AEP3* to growth at 37° might be due to its effect on membrane stability or on transcript stability and translation initiation in the process of energy production in mitochondria. Previous studies identified a possible positive role for mitochondrial integrity and function in growth of yeast at high temperature (Dallabona et al., 2010; Ehrenreich et al., 2010; Parts et al., 2011).

The course of our study was directed toward identifying loci with an over-dominant contribution to heterosis. However, the F2 population and the allele-replacement analyses also demonstrated clearly the contribution of epistasis to heterosis. In the allele-replacement analysis, *AEP3* had an over-dominant effect in the hybrid background but not in the parental backgrounds. To us, this meant that the over-dominance in *AEP3* was contingent upon heterozygosity in other loci, a clear sign of epistasis. In the F2 analyses, we could confirm this finding and point to pairs of interacting loci. We found that *AEP3* and *RRP3* each had epistatic relationship with *Win. 828* and *ADR1.* Eliminating the heterozygotes of the latter two loci changed the contribution of *AEP3* from over-dominant to under-dominant and of *RRP3* from co-dominant to under-dominant by changing the size of the dominant genetic deviation while leaving the additive genetic deviation unchanged. The type of epistasis in these cases indicated that *AEP3* and *RRP3* each had a synergistic interaction with *Win. 828* and *ADR1.* The interactions were synergistic in the sense that a combination of two loci yielded larger d and larger contribution to heterosis than any locus alone. Our study did not look for epistatic interactions in a systematic way, yet within the small collection of five candidates, we found significant evidence for it. Finding significant epistatic effects on the trait among five loci that were identified for a different reason (i.e., for their over-dominant effect) is another piece of evidence for the contribution of these candidates to the trait.

Concerning the genetic models used for explaining heterosis, this study supports the idea that a combination of genetic models underlies this phenotypic phenomenon (Birchler et al., 2010; Baranwal et al., 2012). This idea was indirectly supported also by our previous phenotypic analyses of this hybrid collection (Shapira et al., 2014). So far, over-dominant genes identified in previous studies had a major effect on the hybrid's phenotype (Wallace, 1957; Falk, 1961; Muller and Falk, 1961; Hua et al., 2003; Krieger et al., 2010; Saeed et al., 2011; Li et al., 2013). The over-dominant loci we mapped, including *AEP3,* had a minor effect in the SK1xS1001 background. In QTL mapping studies, loci with major effects on a phenotype are often mapped first, yet, recent advancements made it clear that for several quantitative traits of interest, many additional, or even minor-effect loci exclusively underlie most trait variance (Flint and Mackay, 2009; Mackay et al., 2009; Ehrenreich et al., 2010). Although, identifying over-dominant loci with major effect might be more attractive for utilization in breeding of plants and animals, the method we developed enabled identifying many significant loci, each with a minor effect, even within a specific hybrid background.

Our study suggests that the combined contribution of several minor effect over-dominant and dominant loci might underlie significant heterosis and that is why regarding heterosis as a quantitative phenomenon should be taken into consideration in breeding programs of plants and animals. Additionally, finding interactions between the local genotype of *AEP3* and other loci supports contribution of epistasis to heterosis as was suggested in previous studies (Melchinger et al., 2007, 2008; de Visser et al., 2011; He et al., 2011; Li et al., 2012). Furthermore, while previous work studied either over-dominant or epistatic contributions, our study demonstrated that the contribution of a single locus to heterosis is composed of both over-dominant and epistatic effects. Our findings are taking us additional step further toward understanding of the genetic complexity underlying this extraordinary phenotypic phenomenon and opening an opportunity to apply our methodologies to studies in other species.

## Author contributions

RS and LD conceived the study; LD supervised and provided reagents and infrastructure for the experiments; RS performed the experiments; RS and LD analyzed the data and wrote the manuscript.

## Funding

This research was supported by The Israeli Science Foundation (Grant No. 928/07 to LD).

### Conflict of interest statement

The authors declare that the research was conducted in the absence of any commercial or financial relationships that could be construed as a potential conflict of interest.

## Supplementary material

Supplementary Table 1 **Primers used in this study**.

Supplementary Data Sheet 1 **List of 803 significant heterotic loci**.

Supplementary Data Sheet 2 **Mean normalized DT (h) at 37°C of F2 segregants**.

Supplementary Figure 1 **An illustration of one window that divides the 120 hybrids into three genotypic groups and the distribution of *d*/*a*-values within each group. (A)** Shown is the DNA sequence alignment of the 16 parental strains as in Shapira et al. (2014). Three polymorphic SNPs were found in this window, which divided the 16 strains into two haplotypic groups. Based on their parental haplotypes, the 120 hybrids were divided into three groups: Homozygotes to the first haplotype A, homozygotes to the second haplotype B, and heterozygotes C. **(B)** An example of a comparison between the three genotypic groups in one of the windows. Note the variance around the mean of each group. Each black dot represents one hybrid. The mid-line in each green diamond is the mean of the group and the spread of the diamond is the 95% confidence intervals of the mean.

Supplementary Figure 2 **Distributions of haplotype and window characteristics based on the computational method. (A)** Number of haplotypes per window, **(B)** Number of SNPs per haplotype, and **(C)** Window size in bp.

Supplementary Figure 3 **Doubling time distributions of F1, BC1-S1001 and BC1-SK1 populations at 30° (A) and 37° (B)**. X-axis shows DT-values in hours and Y-axis shows strain counts.
