# Supplementary figures and images for: Genes with a Combination of Over-Dominant and Epistatic Effects Underlie Heterosis in Growth of Saccharomyces cerevisiae at High Temperature
Source: Front Genet. 2016 May 4;7:72. doi: 10.3389/fgene.2016.00072 (PMC4854886; doi:10.3389/fgene.2016.00072)

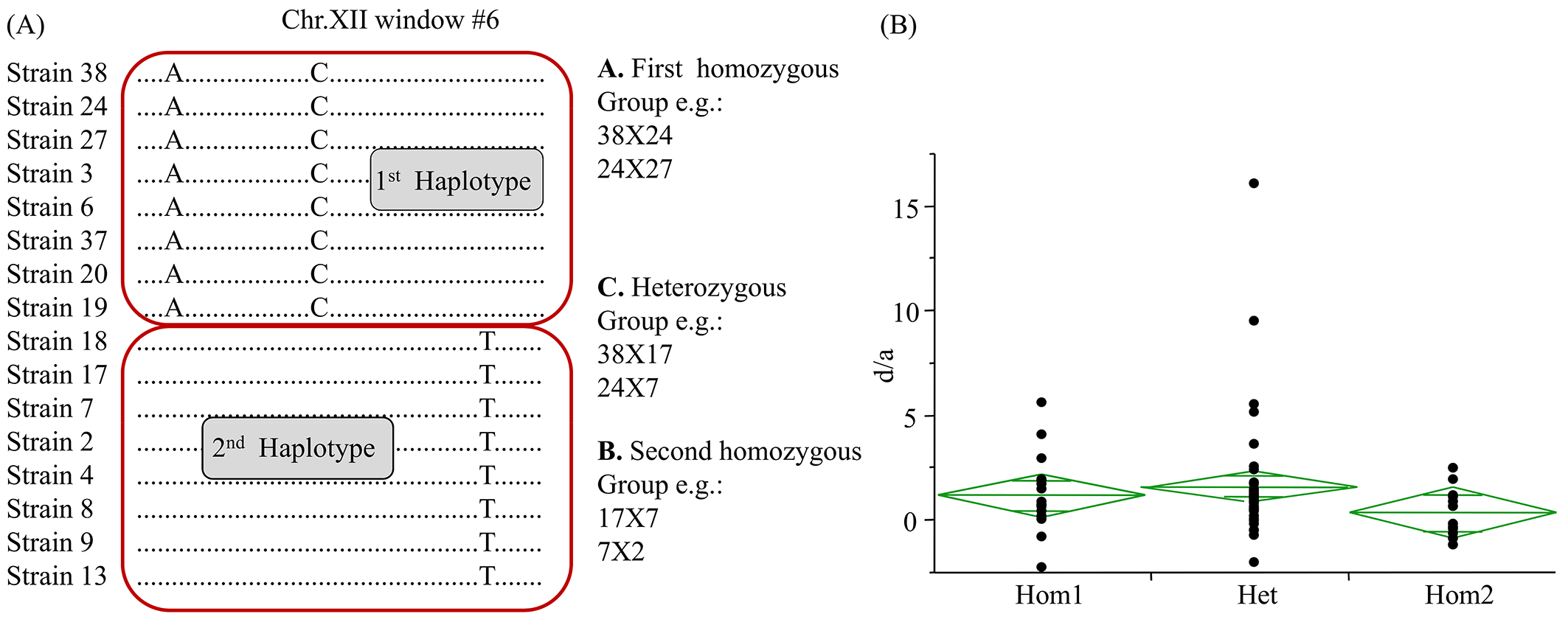

Supplement: Supplementary Figure 1 — An illustration of one window that divides the 120 hybrids into three genotypic groups and the distribution of d/a-values within each group. (A) Shown is the DNA sequence alignment of the 16 parental strains as in Shapira et al. (2014). Three polymorphic SNPs were found in this window, which divided the 16 strains into two haplotypic groups. Based on their parental haplotypes, the 120 hybrids were divided into three groups: Homozygotes to the first haplotype A, homozygotes to the second haplotype B, and heterozygotes C. (B) An example of a comparison between the three genotypic groups in one of the windows. Note the variance around the mean of each group. Each black dot represents one hybrid. The mid-line in each green diamond is the mean of the group and the spread of the diamond is the 95% confidence intervals of the mean. [file Image1.TIF]

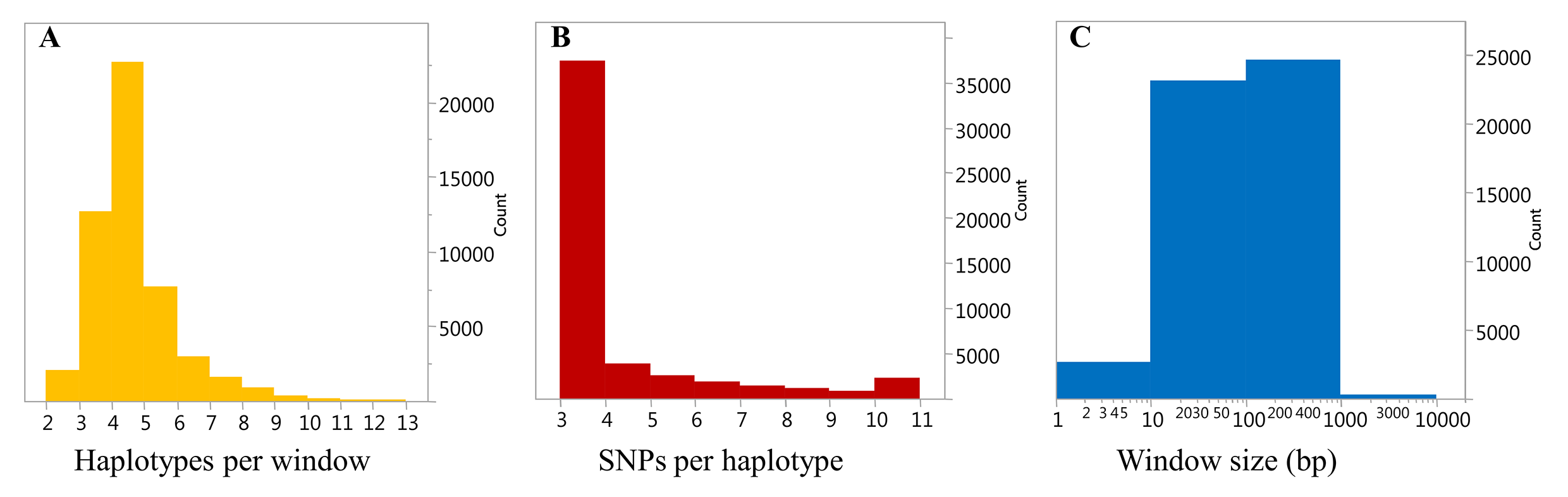

Supplement: Supplementary Figure 2 — Distributions of haplotype and window characteristics based on the computational method. (A) Number of haplotypes per window, (B) Number of SNPs per haplotype, and (C) Window size in bp. [file Image2.TIF]

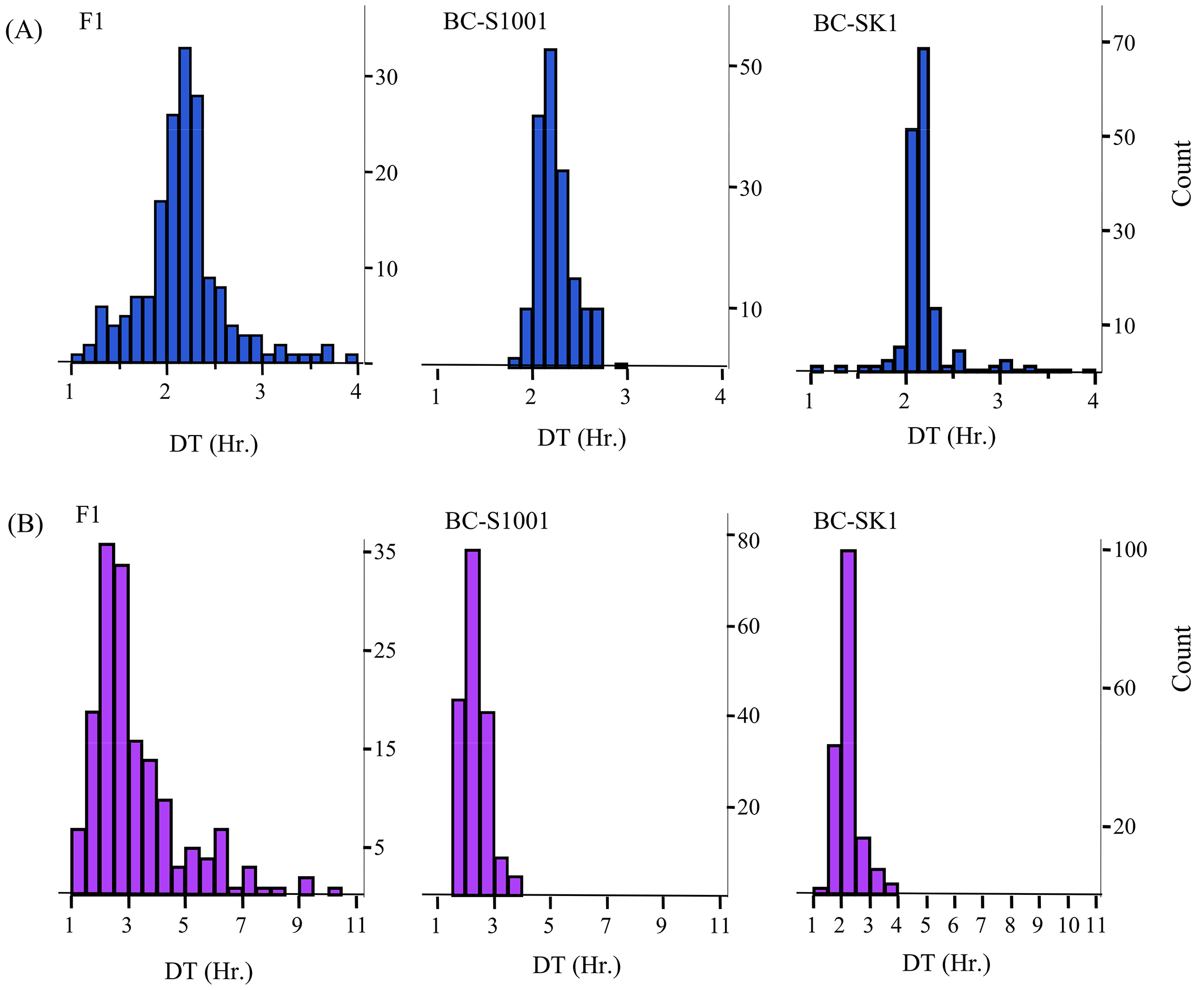

Supplement: Supplementary Figure 3 — Doubling time distributions of F1, BC1-S1001 and BC1-SK1 populations at 30° (A) and 37° (B). X-axis shows DT-values in hours and Y-axis shows strain counts. [file Image3.TIF]
